# Metabolic phenotype in non-aldosterone producing adrenal adenomas with co-existent polycystic ovary syndrome: a joint Ens@t project

**DOI:** 10.1007/s12020-025-04369-7

**Published:** 2025-07-30

**Authors:** Ariadni Spyroglou, Panagiota Konstantakou, Marianna Minnetti, Barbara Altieri, Elisabeth Nowak, Petros Papalexis, Anna Angelousi, Dimitra Vasiliadi, Otilia Kimpel, Djuro Macut, Lorenzo Tucci, Francesca Donnarumma, Guido Di Dalmazi, Theodore Papaioannou, Andrea Isidori, Martin Reincke, Manousos Konstadoulakis, George Mastorakos, Gregory Kaltsas, Krystallenia I. Alexandraki

**Affiliations:** 1 2nd Department of Surgery, Aretaieio Hospital, National and Kapodistrian University of Athens, Athens, Greece; 2 Department of Experimental Medicine, Sapienza University of Rome, Rome, Italy; 3 Division of Endocrinology and Diabetes, Department of Medicine, University Hospital, University of Würzburg, Würzburg, Germany; 4 Department of Medicine IV, LMU University Hospital, LMU Munich, Munich, Germany; 5 First Department of Internal Medicine, Unit of Endocrinology, Laikon General Hospital, National and Kapodistrian University of Athens, Athens, Greece; 6 Department of Endocrinology, Diabetes and Metabolism, European Reference Network on Rare Endocrine Conditions (ENDO-ERN), Evangelismos Hospital, Athens, Greece; 7 Clinic for Endocrinology, Diabetes and Metabolic Diseases University Clinical Centre of Serbia, Faculty of Medicine, University of Belgrade, Belgrade, Serbia; 8 Division of Endocrinology and Diabetes Prevention and Care, IRCCS Azienda Ospedaliero-Universitaria di Bologna, Bologna, Italy; 9 Department of Medical and Surgical Sciences (DIMEC), Alma Mater Studiorum University of Bologna, Bologna, Italy; 10 Department of Biomedical Engineering, Medical School, National and Kapodistrian University of Athens, Athens, Greece; 11 1st Propaedeutic Department of Internal Medicine, National and Kapodistrian University of Athens, Athens, Greece

**Keywords:** Polycystic ovaries syndrome, Non-aldosterone producing adrenal adenoma, Insulin resistance, Hyperandrogenemia, Hypercortisolism

## Abstract

**Purpose:**

Non-aldosterone producing-adrenal-adenomas (NAPACAs) and polycystic ovary syndrome (PCOS) are associated with insulin-resistance (IR). Whether the co-existence of the two diseases leads to accentuated adverse metabolic profile remains unknown. Aim of this study is the assessment of cardiometabolic risk factors in women with NAPACAs with and without PCOS.

**Methods:**

We conducted a retrospective multicenter study including adult premenopausal women categorized as NAPACA (*n* = 45), PCOS (=20) or NAPACA+PCOS (*n* = 24), excluding women with hormonally active adenomas, congenital-adrenal-hyperplasia, diabetes, systemic steroid medication or active malignancy.

**Results:**

NAPACA patients were significantly older than the other two groups (*P* < 0.001). All groups did not differ in blood pressure, HbA1c, fasting plasma glucose (*P* > 0.05) or in body-mass-index (*P* = 0.06). NAPACA+PCOS patients displayed significantly increased insulin resistance (IR) (GIR:*P* < 0.05, HOMA: *P* < 0.05, QUICKI:*P* < 0.05, MATSUDA-index: *P* < 0.05). Cortisol levels upon 1-mg-dexamethasone-suppression-test (DST) did not differ among the groups; DHEA-S (*P* < 0.05) and testosterone (*P* < 0.01) were significantly higher in the two groups with PCOS patients. Free-androgen-index positively correlated with IR in NAPACA (GIR *P* < 0.01, HOMA *P* < 0.05, QUICKI *P* < 0.05) and PCOS (GIR *P* < 0.01, HOMA *P* < 0.01, QUICKI *P* < 0.01, MATSUDA *P* < 0.01), while 1mg-DST positively correlated with IR in NAPACA+PCOS (GIR *P* = 0.05, HOMA *P* < 0.05, QUICKI *P* < 0.05, MATSUDA *P* < 0.05). Younger age, higher IR and lower HDL levels predicted the PCOS presence in NAPACA patients whereas the multivariate analysis revealed age and HDL levels as the most important predictors of this association.

**Conclusion:**

These findings provide evidence for a distinct metabolic pattern in NAPACA+PCOS patients compared to NAPACA patients. Further prospective studies with larger patient cohorts will be necessary to elucidate this observation.

## Introduction

Polycystic ovary syndrome (PCOS), with a prevalence of 6–8%, is the most common endocrinopathy in reproductive-aged women [1] and is characterized by a combination of signs and symptoms of hyperandrogenism and ovarian dysfunction [2, 3]. According to the Androgen Excess and PCOS Society, PCOS is defined by the presence of hyperandrogenism (clinical and/or biochemical), ovarian dysfunction (oligo-anovulation and/or polycystic ovaries), and the exclusion of related disorders, pinpointing the role of androgens on the metabolic risk [4]. PCOS is also strongly associated with obesity and metabolic aberrations including insulin resistance (IR), rendering women with PCOS at increased risk of impaired glucose tolerance and type 2 diabetes mellitus, so that an oral glucose tolerance test (OGTT) should be part of their assessment [2, 5, 6]. Furthermore, these women, regardless of their age and body-mass-index (BMI), are at increased risk of cardiovascular disease [7, 8], so that they should be assessed for their lipid profile and blood pressure levels [7, 9]. From a pathophysiological point of view, IR and its subsequent hyperinsulinemia have been implicated in the development of PCOS through increased luteinizing hormone (LH) pulse and amplitude and, thereby, through stimulation of the ovarian theca cells to synthesize androgens. Vice versa, hyperandrogenism can lead to IR through alterations of insulin action on skeletal muscle and adipose tissue, and through an increase in visceral adiposity [10].

Non-aldosterone producing adrenal adenomas (NAPACA) are benign adrenal adenomas that are not associated with hormonal excess. However, a detailed work-up might reveal biochemical evidence of mild hypercortisolism in patients without clinically overt Cushing syndrome (CS) [11, 12]. Interestingly, an elevated mortality has been documented in NAPACA patients, attributed to mild autonomous cortisol secretion (MACS) by these neoplasms [13, 14]. NAPACA patients, in particular those with MACS or clinically overt CS suffer more frequently from cardiovascular comorbidities, with type 2 diabetes mellitus and dyslipidemia presenting also an increased prevalence in these patients, rendering the cortisol excess a major determinant of IR [14–16]. On the molecular level, elevated insulin levels have been shown to stimulate adrenocortical cell proliferation in vitro, and insulin is acknowledged as a growth factor inducing mass development [17–19]. Whether the subtle cortisol secretion leads to hyperinsulinemia or hyperinsulinemia is the trigger of tumor mass development and subsequent subclinical hypercortisolism remains unclear.

Taken together, it is already acknowledged that both NAPACAs and PCOS are associated with hyperinsulinemia and IR. On the one hand, it has been shown that IR and compensatory hyperinsulinemia induce the development of adrenal masses and PCO. On the other hand, both subtle cortisol overproduction and hyperandrogenemia can lead to IR. Furthermore, both NAPACAs and PCOS exhibit several aspects of the metabolic syndrome, such as obesity, arterial hypertension, dyslipidemia, macro-/microvascular dysfunction, and impaired glucose tolerance or type 2 diabetes mellitus [20]. Whether the co-existence of the two disease entities leads to an accentuated prevalence of the metabolic syndrome or represents an independent disease entity still remains unclear.

The aim of the present study is the assessment of cardiometabolic risk factors and IR indices (IRI) in women with PCOS and with NAPACAs with and without co-existent PCOS.

## Materials and methods

### Patient cohorts

The present study was conducted as part of the European Network for the Study of Adrenal Tumors (ENS@T). The study was conducted according to the Declaration of Helsinki Guidelines and was approved by the Ethics Committee of the Aretaieio Hospital, National and Kapodistrian University of Athens, as coordinating center, and from the respective Ethics Committee of all other participating centers (Decision Nr. 363/13-10-2021). Informed consent was obtained from all patients before study enrollment. The study is presented herein according to the STROBE reporting guidelines. All eligible patients from seven ENS@T centers (4 countries) diagnosed with the above disease entities between January 2000 and July 2024 were retrospectively included in our study. Eligible patients were defined as adult premenopausal women categorized as NAPACA (*n* = 45) or NAPACA+PCOS (*n* = 24), according to the respective definitions of ENS@T/ESE and Endocrine Society guidelines for NAPACA and Androgen and PCOS Society for PCOS [2, 4, 21]. A group of 20 PCOS premenopausal women with normal adrenal glands on recent imaging (computer tomography (CT) or magnetic resonance imaging (MRI)) performed for other reasons was additionally included for comparison reasons. We excluded women with hormonally active adenomas (overt CS, primary aldosteronism or pheochromocytoma), but included patients with MACS (cortisol after 1 mg dexamethasone suppression test (DST) <5 μg/dl), considering cortisol secretion/mild hypersecretion as a continuum with interchangeable metabolic effects. Furthermore, we excluded women with congenital adrenal hyperplasia, diabetes mellitus, systemic steroid medication or active malignancy. Data on their demographic characteristics, clinical, biochemical and hormonal data and glucose and insulin values after a standardized 75 g OGTT were collected. From the recruited patients 3 NAPACA patients had to be excluded as they had type 2 diabetes mellitus, based on their HbA1c or the intake of antidiabetic treatment.

### Clinical and laboratory annotations

Obesity was assessed by the BMI, calculated by the formula: weight (kg) / (height (m))^2^ and by the waist-to-height-ratio (WHR), calculated as: waist circumference (cm) / height (cm). Systolic (SBP, mmHg) and diastolic blood pressure (DBP, mmHg) were measured at the first outpatient visit by a mercury sphygmomanometer with the subject in a seated position, after a rest of at least 5 min. The average of three measurements was obtained. Fasting glucose (GLU, mg/dl), fasting insulin (INS, μU/ml), HbA1c (%), total cholesterol (TC, mg/dl), high-density lipoproteins (HDL, mg/dl), low-density lipoproteins (LDL, mg/dl), triglycerides (TG, mg/dl) levels, as well as cortisol after overnight 1-mg DST (μg/dl), DHEA-S (μg/dl), Δ4-androstenedione (Δ4, ng/ml), testosterone (ng/dl), and sex hormone binding globulin (SHBG, nmol/l) levels were collected. The free androgen index (FAI, %) was calculated as (total Testosterone (nmol/l)/ SHBG (nmol/l)) × 100 [22]. Furthermore, glucose and insulin concentrations from an OGTT with 75 g glucose load performed at 30 min intervals (times 30, 60, 90, 120 min) were determined. The IRI were calculated by the following formulas: Glucose-to-Insulin Ratio (GIR) = fasting GLU/fasting INS; Homeostatic Model assessment for IR (HOMA) index = (fasting GLU (mg/dl) x fasting INS (μU/ml))/405 [23]; Quantitative Insulin Sensitivity Check (QUICKI) index = 1/(log(fasting INS)+log(fasting GLU)) [24]; MATSUDA index = 10000/square root ((fasting GLU × fasting INS) × (mean GLU(OGTT) × mean INS(OGTT))) [25]. NAPACA and NAPACA+PCOS patients had at least one documented adrenal incidentaloma >1 cm in CT or MRI.

### Statistical analysis

Results are reported as mean values ± standard error of the mean (SEM). P-values <0.05 were considered as statistically significant. Normal and log-normal distribution of continuous variables was assessed by applying the non-parametric Kolmogorov–Smirnov test. For normally distributed parameters, an ordinary one-way ANOVA was performed for comparison, whereas a Bonferroni’s multiple comparison test was added in case of significant results. For not normally distributed parameters, the Kruskal-Wallis test was applied, and Dunn’s multiple comparisons test was added in case of significant results. In the case of categorical variables, the chi-square test was applied, whereas for the comparison of two groups the student’s t-test or Mann-Whitney test was used, for normally and non-normally distributed values, respectively. The correlation between the biochemical, hormonal parameters or the IRI and the 1-mg DST, DHEA-S, Δ4-androstenedione and FAI levels, respectively, was evaluated with the Pearson or Spearman rank correlation coefficient, for normally and non-normally distributed values, respectively. Logistic regression analysis was performed to identify clinical and metabolic parameters that were significantly related to the odds of having PCOS in addition to NAPACA. Statistical analysis was performed using GraphPad Prism 9.0.0.

## Results

### Cardiometabolic characteristics of the cohorts

NAPACA patients were significantly older than the other two groups (NAPACA: 40.6 ± 1.1, PCOS: 31.3 ± 1.4, NAPACA+PCOS: 33.8 ± 1.4 years old, *P* < 0.001). PCOS patients displayed the lowest and NAPACA+PCOS the highest BMI (PCOS: 27.1 ± 1.7, NAPACA: 28.2 ± 1.1, NAPACA+PCOS: 31.8 ± 1.9 kg/m^2^), but without a statistically significant difference (*P* = 0.06) and patients of the three groups did not significantly differ in their WHR (NAPACA: 0.533 ± 0.02, PCOS: 0.513 ± 0.02, NAPACA+PCOS: 0.583 ± 0.03, *P* = 0.15). 39% of NAPACA patients had a positive family history of diabetes mellitus type 2, while the percentage increased to 75% for PCOS and to 63% for NAPACA+PCOS patients (*P* = 0.07). Their SBP (NAPACA: 127.9 ± 3.1, PCOS: 128.8 ± 3.0, NAPACA+PCOS: 130 ± 2.6 mmHg, *P* = 0.53) and DBP levels (NAPACA: 81.8 ± 1.8, PCOS: 87.1 ± 3.4, NAPACA+PCOS: 86.5 ± 2.5 mmHg, *P* = 0.19) were comparable in all three groups. Furthermore, all patients had similar HbA1c (NAPACA: 5.335 ± 0.08, PCOS: 5.270 ± 0.05, NAPACA+PCOS: 5.314 ± 0.05%, *P* = 0.64) and fasting glucose levels (NAPACA: 90.3 ± 1.9, PCOS: 95.1 ± 3.8, NAPACA+PCOS: 89.35 ± 1.8 mg/dl, *P* = 0.41). However, NAPACA+PCOS patients displayed significantly higher fasting insulin levels (NAPACA: 9.3 ± 1.0, PCOS: 12.9 ± 2.8, NAPACA+PCOS: 25.5 ± 9.1 μU/ml, *P* = 0.02). NAPACA+PCOS patients also exhibited lower HDL levels (NAPACA: 58.6 ± 2.6, PCOS: 57.3 ± 2.9, NAPACA+PCOS: 48.1 ± 2.5 mg/dl, *P* = 0.02), while their TC (NAPACA: 187.1 ± 4.4, PCOS: 187.7 ± 7.4, NAPACA+PCOS: 191.0 ± 5 mg/dl, *P* = 0.87), LDL (NAPACA: 110.9 ± 4.7, PCOS: 112.7 ± 6.8, NAPACA+PCOS: 119.3 ± 4.3, *P* = 0.54) and TG levels (NAPACA: 101.0 ± 8.7, PCOS: 102.9 ± 15.3, NAPACA+PCOS: 124.7 ± 13.4 mg/dl, *P* = 0.26) did not differ from the other two groups. NAPACA and NAPACA+PCOS patients did not differ in the size of their adrenal incidentaloma (NAPACA: 22.9 ± 2.2, NAPACA+PCOS: 20.0 ± 2.5, *P* = 0.39), (Table 1).

**Table 1:** Baseline parameters of patients with NAPACA, PCOS and NAPACA+PCOS

Parameter	NAPACA	PCOS	NAPACA+PCOS	P-value
Age (years)	40.6 ± 1.1	31.3 ± 1.4	33.8 ± 1.4	<0.001^a^
BMI (kg/m^2^)	28.2 ± 1.1	27.1 ± 1.7	31.8 ± 1.9	0.061
WHR	0.533 ± 0.02	0.513 ± 0.02	0.583 ± 0.03	0.156
Positive family history DM2	39%	75%	63%	0.073
SBP (mmHg)	127.9 ± 3.1	128.8 ± 3.0	130.0 ± 2.6	0.529
DBP (mmHg)	81.8 ± 1.8	87.1 ± 3.4	86.5 ± 2.5	0.192
HbA1c (%)	5.335 ± 0.08	5.270 ± 0.05	5.314 ± 0.05	0.641
GLU (mg/dl)	90.3 ± 1.9	95.1 ± 3.8	89.4 ± 1.8	0.410
INS (μU/ml)	9.3 ± 1.0	12.9 ± 2.8	25.5 ± 9.1	0.022^a^
GIR	13.9 ± 1.4	12.2 ± 1.6	8.3 ± 1.5	0.017^a^
HOMA	2.08 ± 0.26	3.23 ± 0.81	5.75 ± 2.09	0.035^a^
QUICKI	0.358 ± 0.006	0.344 ± 0.009	0.326 ± 0.009	0.018^a^
MATSUDA	6.41 ± 0.8	5.75 ± 0.9	3.57 ± 0.7	0.031^a^
TC (mg/dl)	187.1 ± 4.4	187.7 ± 7.4	191.0 ± 5	0.871
HDL (mg/dl)	58.6 ± 2.6	57.3 ± 2.9	48.1 ± 2.5	0.020^a^
LDL (mg/dl)	110.9 ± 4.7	112.7 ± 6.8	119.3 ± 4.3	0.540
TG (mg/dl)	101.0 ± 8.7	102.9 ± 15.3	124.7 ± 13.4	0.256
1-mg DST (μg/dl)	1.574 ± 0.18	0.829 ± 0.12	1.109 ± 0.19	0.063
DHEA-S (μg/dl)	136.3 ± 26	251.3 ± 42	244 ± 37	0.007^a^
Δ4 (ng/ml)	1.423 ± 0.23	2.684 ± 0.23	2.366 ± 0.35	<0.001^a^
Testosterone (ng/dl)	28.4 ± 2.6	45.2 ± 4.8	44.9 ± 5.2	0.001^a^
FAI	2.93 ± 0.6	4.18 ± 1.0	5.90 ± 1.2	0.015^a^
Adenoma size (mm)	22.9 ± 2.2	—	20.0 ± 2.5	0.393

Data are expressed as mean values±SEM

*BMI* body-mass-index, *1-mg DST* cortisol upon 1 mg dexamethasone suppression test, *DHEA-S* dehydroepiandrosterone sulfate, *DBP* diastolic blood pressure, *DM2* diabetes mellitus type2, *FAI* free androgen index, *GIR* glucose to insulin ratio, *GLU* plasma glucose, *HDL* high-density lipoprotein, *HOMA* homeostatic model assessment for insulin resistance, *INS* insulin, *LDL* low-density lipoprotein, *MACS* mild autonomous cortisol secretion, *MATSUDA* matsuda index, *NAPACA* non-aldosterone producing adrenal adenoma, *PCOS* polycystic ovary syndrome, *QUICKI* quantitative insulin sensitivity check index, *SBP* systolic blood pressure, *TC* total cholesterol, *TG* triglycerides, *WHR* waist to height ratio, *Δ4* Δ4-androstenedione

^a^Denotes statistical significance

### Hormonal profile

When comparing the hormonal profile among the groups, no significant differences were identified in their cortisol levels based on the 1mg-DST (NAPACA: 1.574 ± 0.18, PCOS: 0.829 ± 0.12, NAPACA+PCOS: 1.109 ± 0.19 μg/dl, *P* = 0.06). The DHEA-S (NAPACA: 136.3 ± 26, PCOS: 251.3 ± 42, NAPACA+PCOS: 244 ± 37 μg/dl, *P* = 0.007), Δ4-androstenedione (NAPACA: 1.423 ± 0.23, PCOS: 2.684 ± 0.23, NAPACA+PCOS: 2.366 ± 0.35 ng/ml, *P* < 0.001) and testosterone levels (NAPACA: 28.63 ± 2.6, PCOS: 42.81 ± 4.3, NAPACA+PCOS: 44.93 ± 5.2 ng/dl, *P* = 0.001) were significantly higher in the two groups with PCOS patients. Interestingly, the FAI levels were significantly higher in the NAPACA+PCOS group in comparison to NAPACA (NAPACA: 2.93 ± 0.6, PCOS: 4.18 ± 1.0, NAPACA+PCOS: 5.90 ± 1.2, *P* = 0.015), (Table 1).

### Insulin resistance indices

**Fig. 1:**
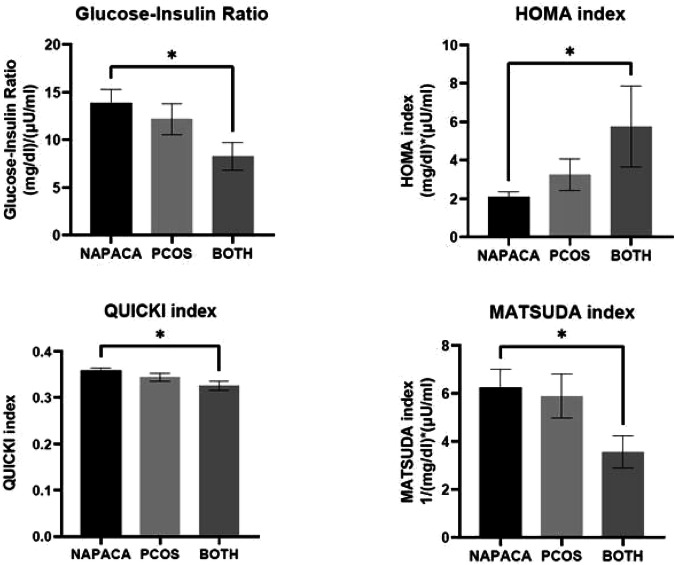
Insulin resistance indices in NAPACA, PCOS and NAPACA+PCOS patients. Upper left panel: Glucose to Insulin Ratio (GIR). Upper right panel: Homeostatic model assessment for insulin resistance (HOMA) index. Lower left panel: Quantitative insulin sensitivity check (QUICKI) index. Lower right panel: MATSUDA index. * denotes statistical significance

We additionally compared both static and dynamic IRI in the three groups. NAPACA+PCOS patients displayed significantly increased IR as calculated by the GIR (NAPACA: 13.9 ± 1.4, PCOS: 12.2 ± 1.6, NAPACA+PCOS: 8.3 ± 1.5, *P* = 0.017), the HOMA index (NAPACA: 2.08 ± 0.26, PCOS: 3.23 ± 0.81, NAPACA+PCOS: 5.75 ± 2.09, *P* = 0 = 035), the QUICKI index (NAPACA: 0.358 ± 0.006, PCOS: 0.344 ± 0.009, NAPACA+PCOS: 0.326 ± 0.009, *P* = 0.018) and the MATSUDA index (NAPACA: 6.41 ± 0.8, PCOS: 5.75 ± 0.9, NAPACA+PCOS: 3.57 ± 0.7, *P* = 0.031) (Fig. 1).

### Correlation of androgens and cortisol to clinical, cardiometabolic parameters and insulin resistance indices

FAI levels positively correlated with insulin levels and the degree of IR in NAPACA (Insulin R 0.54, 95% CI 0.16–0.78, *P* = 0.007, GIR R −0.56, 95% CI −0.79 – −0.19, *P* = 0.005, HOMA R 0.53, 95% CI 0.14–0.77, *P* = 0.008, QUICKI R −0.53, 95% CI −0.77 – −0.14, *P* < 0.05, MATSUDA R −0.49, 95% CI −0.83 – 0.10, *P* = 0.091) and PCOS patients (Insulin R 0.71, 95%CI 0.34 – 0.89, *P* = 0.002, GIR R −0.69, 95%CI −0.88 – −0.30, *P* = 0.003, HOMA R 0.70, 95%CI 0.31–0.89, *P* = 0.002, QUICKI R −0.7, 95% CI −0.89 – −0.31, *P* = 0.002, MATSUDA R −0.82, 95%CI −0.94 – −0.55, *P* = 0.001), (Table 2). Unlike that, cortisol levels after 1mg-DST positively correlated with the degree of IR in the NAPACA+PCOS group (Insulin R 0.51, 95%CI 0.03 – 0.80, *P* = 0.03, GIR R −0.48, 95%CI −0.79 – 0.02, *P* = 0.05, HOMA R 0.58, 95%CI 0.12 – 0.83, *P* = 0.017, QUICKI R −0.58, 95%CI −0.83 – −0.12, *P* = 0.017, MATSUDA R −0.67, 95%CI −0.90 – −0.14, *P* = 0.02) (Table 3). Interestingly, in NAPACA+PCOS patients DHEA-S levels negatively correlated with the BMI (R −0.65, 95%CI −0.85 – −0.28, *P* = 0.002), WHR (R −0.72, 95%CI −0.91 – −0.29, *P* = 0.005), HbA1c levels (R −0.56, 95%CI −0.82 – −0.09, *P* = 0.022) and positively with the HDL levels (R 0.65, 95%CI 0.23 – 0.87, *P* = 0.006), (Table 4). Δ4-androstenedione levels did not present any particular correlation with the cardiometabolic parameters investigated (data not shown).

**Table 2:** Correlation of free androgen index (FAI) to the investigated parameters

FAI	NAPACA			PCOS			NAPACA+PCOS		
	R	95% CI	P	R	95% CI	P	R	95% CI	P
age	−0.348	−0.636–0.025	0.059	0.039	−0.462–0.522	0.881	−0.164	−0.634–0.394	0.555
BMI	0.490	0.139–0.731	0.007^a^	0.468	−0.053–0.788	0.070	0.550	0.036–0.834	0.036^a^
WHR	0.547	0.074–0.819	0.025^a^	0.407	−0.149–0.768	0.133	0.323	−0.494–0.837	0.435
SBP	0.396	0.030–0.668	0.030^a^	0.383	−0.155–0.746	0.143	0.071	−0.513–0.612	0.821
DPB	0.315	−0.062–0.614	0.090	0.337	−0.206–0.721	0.200	−0.193	−0.683–0.416	0.527
HbA1c	0.146	−0.267–0.514	0.478	0.598	0.149–0.842	0.013^a^	0.681	0.190–0.899	0.012^a^
GLU	−0.079	−0.442–0.306	0.683	0.426	−0.084–0.759	0.089	0.491	−0.071–0.816	0.077
INS	0.535	0.155–0.777	0.007^a^	0.711	0.336–0.891	0.002^a^	0.525	−0.090–0.850	0.084
GIR	−0.556	−0.789– −0.185	0.005^a^	−0.689	−0.882– −0.297	0.003^a^	−0.497	−0.839–0.127	0.104
HOMA	0.526	0.144–0.772	0.008^a^	0.699	0.314–0.886	0.002^a^	0.559	−0.041–0.863	0.063
QUICKI	−0.526	−0.772– −0.144	0.008^a^	−0.699	−0.886– −0.314	0.002^a^	−0.559	−0.863–0.041	0.063
MATSUDA	−0.490	−0.826–0.102	0.091	−0.821	−0.935– −0.552	0.001^a^	−0.539	−0.855–0.071	0.075
TC	−0.002	−0.372–0.368	0.991	0.131	−0.386–0.586	0.613	0.033	−0.541–0.586	0.921
HDL	−0.503	−0.736–−0.163	0.005^a^	−0.564	−0.833–−0.079	0.025^a^	0.126	−0.497–0.664	0.695
LDL	0.176	−0.208–0.512	0.353	0.109	−0.422–0.584	0.686	−0.042	−0.614–0.559	0.899
TG	0.249	−0.134–0.566	0.185	0.377	−0.162–0.742	0.151	0.324	−0.293–0.751	0.280
1-mg DST	−0.055	−0.422–0.328	0.778	−0.262	−0.816–0.543	0.531	0.330	−0.319–0.768	0.293
DHEA−S	0.262	−0.205–0.631	0.251	0.403	−0.132–0.756	0.123	0.095	−0.473–0.606	0.750
Δ4	0.375	−0.057–0.689	0.075	0.371	−0.169–0.739	0.158	0.181	−0.426–0.676	0.554

*BMI* body-mass-index, *1-mg DST* cortisol upon 1 mg dexamethasone suppression test, *DHEA-S* dehydroepiandrosterone sulfate, *DBP* diastolic blood pressure, *FAI* free androgen index, *GIR* glucose to insulin ratio, *GLU* plasma glucose, *HDL* high-density lipoprotein, *HOMA* homeostatic model assessment for insulin resistance, *INS* insulin, *LDL* low-density lipoprotein, *MATSUDA* matsuda index, *NAPACA* non-aldosterone producing adrenal adenoma, *PCOS* polycystic ovary syndrome, *QUICKI* quantitative insulin sensitivity check index, *SBP* systolic blood pressure, *TC* total cholesterol, *TG* triglycerides, *WHR* waist to height ratio, *Δ4* Δ4-androstenedione

^a^Denotes statistical significance

**Table 3:** Correlation of cortisol after 1 mg dexamethasone suppression test to the investigated parameters

1mg DST	NAPACA			PCOS			NAPACA+PCOS		
	R	95% CI	P	R	95% CI	P	R	95% CI	P
age	0.483	0.205–0.689	0.001^a^	−0.252	−0.761–0.449	0.482	0.236	−0.244–0.623	0.317
BMI	0.157	−0.172–0.454	0.333	−0.416	−0.864–0.343	0.266	0.561	0.145–0.809	0.010^a^
WHR	−0.077	−0.484–0.358	0.728	−0.361	−0.850–0.461	0.380	0.267	−0.300–0.694	0.333
SBP	0.149	−0.180–0.448	0.359	−0.447	−0.876–0.376	0.267	0.085	−0.410–0.541	0.738
DPB	0.103	−0.225–0.410	0.528	−0.506	−0.893–0.308	0.200	−0.016	−0.492–0.466	0.950
HbA1c	0.245	−0.101–0.538	0.150	−0.264	−0.766–0.437	0.461	0.255	−0.271–0.664	0.320
GLU	0.345	0.036–0.593	0.025^a^	−0.123	−0.698–0.550	0.736	0.417	−0.061–0.739	0.076
INS	0.080	−0.275–0.416	0.650	−0.286	−0.776–0.419	0.423	0.514	0.029–0.803	0.037^a^
GIR	−0.003	−0.350–0.350	0.988	−0.042	−0.654–0.604	0.909	−0.478	−0.786–0.018	0.054^a^
HOMA	0.114	−0.243–0.444	0.519	−0.307	−0.785–0.400	0.389	0.579	0.121–0.834	0.017^a^
QUICKI	−0.114	−0.444–0.243	0.519	0.050	−0.598–0.659	0.890	−0.579	−0.834–0.121	0.017^a^
MATSUDA	−0.290	−0.675–0.218	0.242	−0.047	−0.657–0.601	0.898	−0.671	−0.902–0.138	0.020^a^
TC	−0.046	−0.353–0.270	0.773	−0.068	−0.669–0.587	0.851	0.139	−0.364–0.579	0.583
HDL	−0.153	−0.444–0.167	0.333	−0.005	−0.633–0.627	0.989	−0.257	−0.655–0.252	0.303
LDL	0.068	−0.250–0.373	0.669	−0.226	−0.749–0.471	0.531	0.271	−0.255–0.674	0.290
TG	0.119	−0.201–0.416	0.454	0.202	−0.490–0.738	0.575	0.498	0.026–0.789	0.035^a^
DHEA-S	−0.588	−0.796– −0.26	0.001^a^	−0.639	−0.915–0.043	0.064	−0.284	−0.692–0.261	0.283
Δ4	−0.311	−0.607–0.059	0.088	0.263	−0.486–0.789	0.494	−0.473	−0.776–0.007	0.047
FAI	−0.055	−0.422–0.328	0.778	−0.262	−0.816–0.543	0.531	0.330	−0.319–0.768	0.293

*BMI* body-mass-index, *1-mg DST* cortisol upon 1 mg dexamethasone suppression test, *DHEA-S* dehydroepiandrosterone sulfate, *DBP* diastolic blood pressure, *FAI* free androgen index, *GIR* glucose to insulin ratio, *GLU* plasma glucose, *HDL* high-density lipoprotein, *HOMA* homeostatic model assessment for insulin resistance, *INS* insulin, *LDL* low-density lipoprotein, *MATSUDA* matsuda index, *NAPACA* non-aldosterone producing adrenal adenoma, *PCOS* polycystic ovary syndrome, *QUICKI* quantitative insulin sensitivity check index, *SBP* systolic blood pressure, *TC* total cholesterol, *TG* triglycerides, *WHR* waist to height ratio, *Δ4* Δ4-androstenedione

^a^Denotes statistical significance

**Table 4:** Correlation of DHEA-S to the investigated parameters

DHEA-S	NAPACA			PCOS			NAPACA+PCOS		
	R	95% CI	P	R	95% CI	P	R	95% CI	P
age	−0.647	−0.826– −0.352	<0.001^a^	0.214	−0.311–0.639	0.407	−0.412	−0.730–0.051	0.071
BMI	−0.320	−0.642–0.098	0.119	0.282	−0.263–0.691	0.289	−0.654	−0.854– −0.284	0.002^a^
WHR	−0.312	−0.731–0.278	0.275	0.411	−0.145–0.770	0.130	−0.719	−0.908– −0.290	0.005^a^
SBP	0.223	−0.183–0.564	0.264	0.206	−0.337–0.646	0.441	−0.183	−0.609–0.323	0.466
DPB	0.046	−0.350–0.429	0.819	0.373	−0.167–0.740	0.155	−0.089	−0.545–0.406	0.724
HbA1c	−0.392	−0.699–0.037	0.064	0.340	−0.183–0.713	0.181	−0.556	−0.823– −0.088	0.022^a^
GLU	−0.147	−0.508–0.258	0.466	0.200	−0.324–0.631	0.438	0.229	−0.265–0.628	0.346
INS	−0.080	−0.515–0.388	0.738	0.132	−0.385–0.587	0.612	−0.134	−0.588–0.384	0.607
GIR	0.079	−0.389–0.514	0.741	−0.056	−0.534–0.449	0.832	0.092	−0.420–0.559	0.724
HOMA	−0.087	−0.520–0.382	0.714	0.125	−0.392–0.582	0.632	−0.150	−0.598–0.370	0.564
QUICKI	0.087	−0.382– 0.520	0.714	−0.125	−0.582–0.392	0.632	0.150	−0.370–0.598	0.564
MATSUDA	0.651	−0.199–0.942	0.113	−0.179	−0.617–0.344	0.491	−0.223	−0.670–0.341	0.421
TC	−0.123	−0.489–0.280	0.542	0.042	−0.460–0.523	0.874	0.041	−0.447–0.509	0.874
HDL	0.125	−0.279–0.491	0.535	0.090	−0.438–0.572	0.740	0.649	0.231–0.865	0.006^a^
LDL	−0.254	−0.586–0.151	0.201	−0.240	−0.657–0.320	0.401	−0.124	−0.581–0.393	0.999
TG	−0.352	−0.652–0.045	0.072	0.156	−0.382–0.615	0.561	−0.499	−0.789–0.027	0.035^a^
1-mg DST	−0.588	−0.796– –0.26	0.001^a^	−0.639	−0.915–0.043	0.064	−0.284	−0.692–0.261	0.283
Δ4	0.657	0.261–0.864	0.003	0.224	−0.321–0.657	0.404	0.596	0.147–0.842	0.013^a^
FAI	0.262	−0.205–0.631	0.251	0.403	−0.132–0.756	0.123	0.095	−0.473–0.606	0.750

*BMI* body-mass-index, *1-mg DST* cortisol upon 1 mg dexamethasone suppression test, *DHEA-S* dehydroepiandrosterone sulfate, *DBP* diastolic blood pressure, *FAI* free androgen index, *GIR* glucose to insulin ratio, *GLU* plasma glucose, *HDL* high-density lipoprotein, *HOMA* homeostatic model assessment for insulin resistance, *INS* insulin, *LDL* low-density lipoprotein, *MATSUDA* matsuda index, *NAPACA* non-aldosterone producing adrenal adenoma, *PCOS* polycystic ovary syndrome, *QUICKI* quantitative insulin sensitivity check index, *SBP* systolic blood pressure, *TC* total cholesterol, *TG* triglycerides, *WHR* waist to height ratio, *Δ4* Δ4-androstenedione

^a^Denotes statistical significance

A logistic regression analysis was performed to provide the clinical and/or cardiometabolic predictors in the NAPACA and NAPACA+PCOS groups. In the univariate analysis, age (OR: 0.887; 95%CI: 0.823–0.995, *P* = 0.002), the MATSUDA index (OR: 0.728; 95%CI: 0.555–0.954, *P* = 0.02) and HDL levels (OR: 0.944; 95%CI: 0.900–0.991, *P* = 0.02) predicted the presence of PCOS in NAPACA patients, while, in the multivariate analysis, only the younger age and the lower HDL levels predicted the PCOS presence (age: OR: 0.767; 95%CI: 0.620–0.949, *P* = 0.014; HDL: OR: 0.911, 95%CI: 0.833–0.997, *P* = 0.04) (Table 5).

**Table 5:** OR and 95% CI for each metabolic parameter to distinguish NAPACA from NAPACA+PCOS

	Univariate analysis			Multivariate analysis		
Parameter	OR	95% CI	P	OR	95% CI	P
Age	0.887	0.823–0.955	0.002^a^	0.767	0.620–0.949	0.014^a^
BMI	1.056	0.990–1.127	0.097			
SBP	1.007	0.978–1.037	0.646			
MATSUDA	0.728	0.555–0.954	0.021^a^	0.932	0.668–1.300	0.679
HDL	0.944	0.900–0.991	0.020^a^	0.911	0.833–0.997	0.042^a^
Size	0.977	0.936–1.020	0.284			

For the ORs, numerators are the odds in the NAPACA+PCOS group (group 1), denominators are the odds in the NAPACA group (group 0)

^a^Denotes statistical significance

As our cohort included patients with MACS, 12 in the NAPACA group (27%), and one in the NAPACA+PCOS group (5%), we further performed a nested analysis, excluding all MACS patients and reassessing the IRI in all groups. NAPACA+PCOS patients still displayed significantly increased IR as calculated by the GIR (NAPACA: 13.5 ± 1.4, PCOS: 12.6 ± 1.7, NAPACA+PCOS: 8.5 ± 1.5, *P* = 0.025), the HOMA index (NAPACA: 1.9 ± 0.25, PCOS: 3.17 ± 0.85, NAPACA+PCOS: 5.70 ± 2.2, *P* = 0.05), the QUICKI index (NAPACA: 0.358 ± 0.006, PCOS: 0.346 ± 0.009, NAPACA+PCOS: 0.327 ± 0.01, *P* = 0.03) and the MATSUDA index (NAPACA: 6.67 ± 0.8, PCOS: 5.9 ± 0.9, NAPACA+PCOS: 3.7 ± 0.7, *P* = 0.03). HDL levels remained lowest in the NAPACA+PCOS group (NAPACA: 61.1 ± 3.1, PCOS: 57.3 ± 2.9, NAPACA+PCOS: 48.5 ± 2.6, *P* = 0.02). Similarly, uni- and multivariate analysis of these nested groups also denoted age and HDL as the major predictors of the presence of PCOS in NAPACA patients [univariate analysis: age (OR: 0.916; 95%CI: 0.848–0.991, *P* = 0.028), the MATSUDA index (OR: 0.702; 95%CI: 0.495–0.995, *P* = 0.047) and HDL levels (OR: 0.943; 95% CI 0.892–0.996, *P* = 0.037); multivariate analysis: age (OR: 0.721; 95%CI 0.535–0.972, *P* = 0.032) and HDL (OR: 0.887; 95%CI 0.776–1.014, *P* = 0.079)].

## Discussion

In the present study we compared the clinical, biochemical and hormonal parameters related to an adverse metabolic profile among patients with NAPACA, PCOS or NAPACA+PCOS and identified a distinct pattern of adverse metabolic phenotype in NAPACA+PCOS patients compared to either NAPACA or PCOS patients. This is, to the best of our knowledge, the first study investigating the degree of IR in this patients’ group since little has so far been described in patients with co-existence of NAPACA and PCOS.

In our cohort, NAPACA patients were significantly older than the other two groups, as expected by the natural history of the appearance of adrenal incidentalomas. Interestingly, though, women with NAPACA+PCOS were comparably young to PCOS patients, rendering this group distinct from older typical NAPACA patients. In addition, NAPACA+PCOS tended to display higher BMI and WHR levels than the other two groups, this finding did not reach statistical significance. Several studies have previously acknowledged the presence of obesity in NAPACA patients, as well as the remission of obesity after adrenalectomy [17, 26–28]. In the case of PCOS, obesity characterizes only in part PCOS patients and correlates with a worse metabolic profile but lower adrenal activity, related to DHEA-S levels [29, 30].

In our study, the three groups did not differ in their glucose or HbA1c levels, but NAPACA+PCOS patients had significantly higher fasting insulin levels, suggesting hyperinsulinemia and subsequent IR, the major discerning trait from the other two groups. In line with this observation, we demonstrated a significantly higher IR in NAPACA+PCOS patients quantified by both static (GIR, HOMA index, QUICKI index) as well as dynamic (MATSUDA index) IRI. Similarly, a previous study in adrenal adenoma patients documented a pronounced IR of all adenoma patients investigated and hypothesized that adrenal incidentalomas are a manifestation of the metabolic syndrome occurring through a mechanism reminiscent of the effect of insulin on ovarian stimulation observed in PCOS [17]. In this context a further study had documented that NAPACA patients exhibited higher fasting glucose, insulin, and HOMA index, with reduced MATSUDA index [31]. Unlike that, a study by Terzolo et al., did not identify any differences in the static IRI in NAPACA patients in comparison to controls but recognized a worse response to an OGTT in NAPACA patients [15]. Similarly, Anderwald et al., observed muscle IR after OGTT in NAPACA patients but no changes in their baseline insulin sensitivity [16]. Papanastasiou et al. observed additionally a deterioration of the IR in NAPACA patients in the 5-year follow-up following an incremental continuum trend from normal to increased cortisol secretion [32]. Interestingly, in our present analysis, also when excluding MACS, the IR remained significantly higher in the NAPACA+PCOS group, suggesting that the continuum of cortisol secretion levels is not the sole factor affecting IR. On the other hand, hyperinsulinemia and IR are well acknowledged common traits of PCOS, affecting both obese and to a lesser extent non-obese PCOS patients [33]. Interestingly, though, herein NAPACA+PCOS patients display more extensive IR than each individual group of NAPACA or PCOS patients, suggesting either the contribution of two different pathogenetic mechanisms in the generation of IR or an additive effect in the presence of the two disease entities implying a distinct trait in the group displaying both entities simultaneously.

The investigation of further parameters of the metabolic syndrome in our cohort did not reveal any significant differences in the SBP and DBP levels between the studied groups, unlike two previous studies documenting increased blood pressure levels in patients with non-functioning adrenal adenomas, studies that, however, did not discern between truly non-functioning adenomas and MACS [15, 31]. From the lipid parameters studied herein, NAPACA+PCOS patients showed significantly lower HDL levels, in line with their aggravated metabolic profile, similar to a previous study [31]. Terzolo et al., however, did not document any alterations in the lipid profile of patients with adrenal adenomas, including also patients with subclinical hypercortisolism in their cohort [15]. Also, PCOS patients display an adverse lipid profile, with increased LDL/HDL levels, that can partially be reversed with antiandrogen treatment [34]. In our study, in the multivariate analysis, higher HDL levels together with an older age were the characteristics discerning NAPACAs from NAPACAS+PCOS. Regarding the adenomas, in our study we did not identify any differences in the adenoma size between NAPACA and NAPACA+PCOS patients, whereas previously, the size of adrenal incidentalomas had been correlated with the degree of IR [35].

Concerning the hormonal profile of the three groups investigated herein, no differences were observed in their cortisol levels after DST, even when the presence of MACS was more frequent in the NAPACA group. As expected, the groups including PCOS displayed significantly higher androgen levels, without differences between PCOS or NAPACA+PCOS patients. However, the FAI levels positively correlated with the degree of IR in both NAPACA and PCOS groups, whereas the cortisol levels after DST positively correlated with the degree of IR in NAPACA+PCOS patients, suggesting a possibly synergistic effect of androgens and cortisol on the reduction of insulin sensitivity in this group. In a previous study, it was hypothesized that patients with apparently non-functioning adrenal adenomas might be at an increased cardiovascular risk due to increased cortisol secretion [36]. A previous study on adrenal adenoma patients had shown that post-OGTT, these patients display cortisol hyperresponsiveness [37]. On the other hand, a study on PCOS presented a partial reversion of IR in PCOS patients under antiandrogen treatment, in line with our observation on the positive correlation of IR with the FAI [38]. Still, in our NAPACA group, no clear hyperandrogenism was documented, suggesting possibly an only subtle effect of the androgens on hyperinsulinemia.

To the best of our knowledge this is the first retrospective multicenter study discussing the metabolic phenotype in co-existence of NAPACA and PCOS and we demonstrate a unique adverse metabolic pattern in this disease entity. The retrospective design of our study and the relatively small cohort size, particularly of the NAPACA+PCOS group, might limit the generalizability of the conclusions, so that future prospective studies are required to validate our findings. A further limitation of our study is the fact that the investigated cohorts differed in age, but this fact may be explained by the natural history of the development of adrenal incidentalomas, that usually occur at an older age. Still, the main differences were observed in the NAPACA+PCOS patients, diming the effect of this bias. In a similar way, the statistically significant presence of MACS in the NAPACA group did not present an interference with the IR results that were more pronounced in the NAPACA+PCOS group.

## Conclusion

In this retrospective multicenter study, the distinct adverse metabolic pattern of patients with co-existent NAPACA+PCOS was characterized in detail. We documented increased IR both under baseline conditions and after an OGTT in NAPACA+PCOS patients when they were compared with NAPACA only patients and PCOS only patients with comparable BMI, glucose, HbA1c, blood pressure and incidentaloma size in the case of NAPACA presence. Although IR positively correlated with the FAI in NAPACA and in PCOS patients, in NAPACA+PCOS all IRI correlated with cortisol levels after DST. We hypothesize a synergistic effect of androgens and cortisol on the generation of this metabolic profile, with a continuum of the metabolic burden from NAPACA without PCOS to NAPACA with PCOS. Further larger prospective multicenter studies will be necessary to elucidate the role of androgens and cortisol on the NAPACA+PCOS metabolic phenotype. Similarly, such studies could additionally provide information on the prevalence of NAPACA in PCOS patients, and investigate whether older age or a PCOS group sharing particular characteristics is more prone to the development of NAPACA.

## Data Availability

The data that support the findings of this study are available from the corresponding author upon reasonable request.
